# Analysis and Optimization of Four-Coil Planar Magnetically Coupled Printed Spiral Resonators

**DOI:** 10.3390/s16081219

**Published:** 2016-08-03

**Authors:** Sadeque Reza Khan, GoangSeog Choi

**Affiliations:** Department of Information and Communication Engineering, SoC Design Laboratory, Chosun University, Gwangju 61452, Korea; sadeque_008@yahoo.com

**Keywords:** bio-implantable devices, magnetic resonance coupling, power delivered to the load, power-transfer efficiency, quality factor

## Abstract

High-efficiency power transfer at a long distance can be efficiently established using resonance-based wireless techniques. In contrast to the conventional two-coil-based inductive links, this paper presents a magnetically coupled fully planar four-coil printed spiral resonator-based wireless power-transfer system that compensates the adverse effect of low coupling and improves efficiency by using high quality-factor coils. A conformal architecture is adopted to reduce the transmitter and receiver sizes. Both square architecture and circular architectures are analyzed and optimized to provide maximum efficiency at a certain operating distance. Furthermore, their performance is compared on the basis of the power-transfer efficiency and power delivered to the load. Square resonators can produce higher measured power-transfer efficiency (79.8%) than circular resonators (78.43%) when the distance between the transmitter and receiver coils is 10 mm of air medium at a resonant frequency of 13.56 MHz. On the other hand, circular coils can deliver higher power (443.5 mW) to the load than the square coils (396 mW) under the same medium properties. The performance of the proposed structures is investigated by simulation using a three-layer human-tissue medium and by experimentation.

## 1. Introduction

Wireless power-transmission systems based on inductive coupling are extensively used in implantable microelectronic devices to increase patient’s comfort and reduce the risk of infection due to the use of transcutaneous wires or the chemical side effects of a battery. The size and lifetime of the battery is another issue of concern in powering bio-implantable devices. The smallest possible size is a major requirement for bio-implants. Depending on its size, the functional depth of an implantable device in a human biological tissue is determined. Usually, these devices are placed in less than 10 mm of depth in human tissue. General implanted microsystems are placed at 1–4 mm of depth. Cochlear implants need to be placed at 3–6 mm inside the temporal bone, and for retinal implants, the expected depth is 5 mm [1,2,3].

The applications of wireless power transmission span over a broad range from submicrowatt to few kilowatts [4,5,6,7,8,9,10,11,12]. Inductive-coupling-based wireless power transfer (WPT) systems are commonly used for implantable devices [13,14,15,16,17,18]. The primary and secondary coils are used as the transmitter (TX) and receiver (RX), respectively, in a two-coil-based inductively coupled WPT. A two-coil-based WPT system suffers from low quality factor (Q-factor or Q) and low coupling coefficient (*k*) due to the source and load resistances. Hence, the maximum achievable power-transfer efficiency (PTE) is relatively low in such systems [19,20,21]. Moreover, most of the previous coils are designed based on filament or Litz wire. The manufacturing and reduction of the size of such coils require proper expertise and sophisticated machinery. As a result, planar and lithographically defined printed spiral coils (PSCs) were introduced in [22]. Flexibility and geometric compactness make the PSC suitable for biomedical devices. 

The PTE of a WPT system depends on the architecture (dimension and structure) of the coils, physical spacing between the coils, relative location, and environment of the coils. The mutual spacing between the TX (or external coil) and RX (or implanted coil) of a biomedical implant must be smaller than the wavelength in the near field and it depends on the dimensions of the coils. The implanted coil must be carefully designed as it is located inside the human body, and it should be as small as possible.

**Figure 1:**
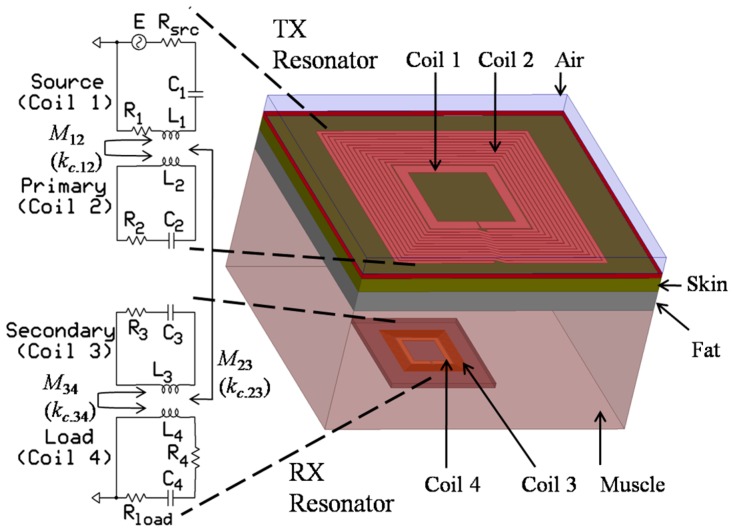
Simplified physical and electrical configuration of a fully planar WPT in a human tissue medium.

An efficient power-transfer method is required for bio-implants to satisfy the power specification and safety issues. Thus, resonant-based power delivery technique has become popular nowadays. This WPT technique typically uses four coils (source, primary, secondary, and load) [23]. The source and primary coils as well as the secondary and load coils are coupled by inductive link. In the TX side, a pair of coils, which are referred to as the source (coil 1 or C1) and primary (coil 2 or C2) coils is used, shown in Figure 1. Another pair of coils, which are referred to as the secondary (coil 3 or C3) and load (coil 4 or C4) coils is used on the RX side. All the coils are tuned at the same resonance frequency (*f_res_*) by *C*_1_–*C*_4_ capacitors. *R*_1_–*R*_4_ are parasitic resistances. *L_i_*, where *i* = 1 to 4, is the self-inductance of the coil. *M_ij_* and *k_c_*_.*ij*_ represent the mutual inductance and coupling coefficient, respectively, of two adjacent coils. In four-coil-based WPT, the TX coil stores energy in the same way as a discrete LC tank. TX coil can be modeled as a tuned step-up transformer as well, where the source can be represented as a primary of the transformer and it can be connected to the source through a power amplifier. The primary of the TX coil can be modeled as the secondary of the transformer which is left open. The RX functions in a similar manner as a step-down transformer. In contrast with the two-coil-based WPT, the effect of source and load resistance can be reduced by the C1 and C4, respectively, in a four-coil WPT, thus can enhance the overall PTE. In a four-coil-based WPT, a high matching condition can be achieved by adjusting the coupling coefficient between source-primary and secondary-load coils. Therefore, it is possible to transfer high power to the load. The distance between TX and RX is 10–20 mm, and the separation medium is human biological tissue. The architecture of the TX and RX coils shown in Figure 1 is conformal [24,25,26] or fully planar [27]. This structure eliminates the possibility of misalignment between coil 1 and coil 2 as well as between coil 3 and coil 4 [28]. Thus, this method improves the overall PTE under misalignment conditions. 

In this paper, a fully planar four-coil magnetic resonance coupling (MRC)-based WPT is analyzed using circuit-based modeling. Both square and circular architectures are considered for analysis and optimization for asymmetrical spiral inductors. The reflected-load theory [29] is used to analyze the Q-factor and power delivered to the load (PDL). The accuracy of the design model is improved by considering the effects of parasitic components as well as the surrounding environments. Critical design parameters, including the TX and RX dimensions, number of turns of the PSC, self- and mutual inductances, coupling coefficients, and Q-factors, are analytically investigated, and the optimum design is determined by applying the design constraints through a step-by-step procedure in MATLAB. A simulation-based comparative study is done on the PTE of the MRC-WPT of both square and circular resonators in air and the human-tissue media. Effect of misalignment on over-all PTE is characterized and measured as well.

This paper is organized as follows: Section 2 formulates the theoretical modeling of the MRC-WPT system. Section 3 describes the optimization method of the PSCs using an iterative approach by utilizing the theoretical model of Section 2 in MATLAB. Section 4 presents the simulation results, experimental setup, and necessary comparisons of the results. 

## 2. Theoretical Modeling of MRC-WPT System

**Figure 2:**
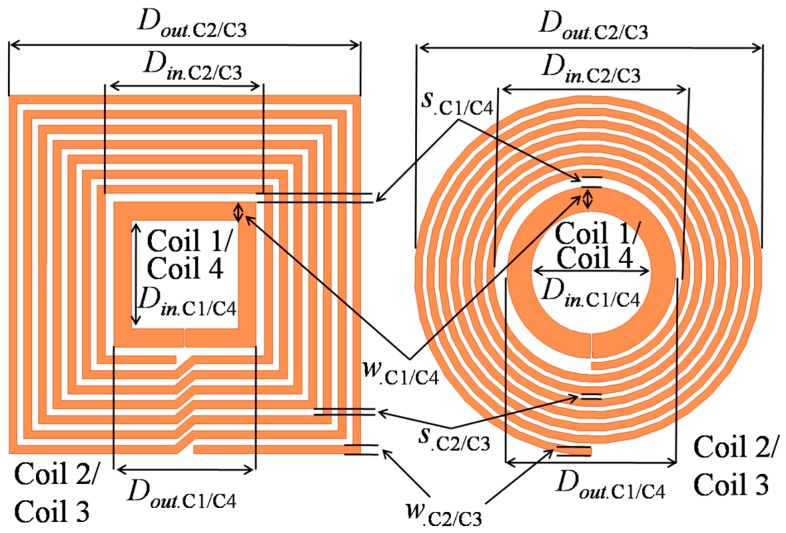
Geometrical architecture of the square- and circular-shaped resonators.

This section illustrates the individual models for the self- and mutual inductance, parasitic components, and Q-factor. The models are based on both square and circular coils in the PSC architecture. A detailed analysis of the PTE and PDL is presented on the basis of the accurate equivalent circuit model of the proposed MRC-WPT system. Figure 2 shows the proposed planar MRC-WPT architecture and the geometrical parameters of both the square- and circular-shaped coils. In Figure 2, coil 1 or coil 4 represents the source or load coil, respectively, and coil 2 or coil 3 represent the primary or secondary coil, respectively. *D_in._*_C*x/y*_ and *D_out._*_C*x/y*_ denote the inner and outer diameters, respectively. In addition, another two important parameters *w_._*_C*x/y*_ and *s_._*_C*x/y*_ represent the line width and spacing, respectively. The subscript “C*x/y*” corresponds to a particular coil from C1 to C4.

### 2.1. Inductance Modeling

Self-inductance *L* is a function of the magnetic flux of the current-carrying conductor and the amount of current flowing through the conductor. The sides of the spirals are approximated to be symmetrical current sheets to measure the self-inductance. Adjacent sheets are considered as orthogonal with zero mutual inductance. On the other hand, the current-carrying opposite sheets have mutual inductance *M*. Equation (1) expresses the simplified self-inductance, which is estimated using the concept of geometric mean, arithmetic mean, and arithmetic mean square distances [30]:

(1) $$L=\frac{C_{l1}\mu_{0}n_{.Cx}^{2}\left(\frac{D_{out.Cx}+D_{in.Cx}}{2}\right)}{2}\;\left[\ln\left(\frac{C_{l2}}{\varphi }\right)+C_{l3}\varphi +C_{l4}\varphi^{2}\right]$$

where *ϕ = (D_out._*_C*x*_ − *D_in._*_C*x*_*)/(D_out._*_C*x*_* + D_in._*_C*x*_*)*, which is the fill factor, and *C_li_* is the layout-dependent coefficient. For circular coils, *C_l_*_1_ = 1, *C_l_*_2_ = 2.46, *C_l_*_3_ = 0.0, and *C_l_*_4_ = 0.2. For square coils, *C_l_*_1_ = 1.27, *C_l_*_2_ = 2.07, *C_l_*_3_ = 0.18, and *C_l_*_4_ = 0.13. In Equation (1), *n_._*_C*x*_ represents the number of PSC turns of coil C*x* (where *x* = 1, 2, 3, and 4).

For unity-turn coils, the Neumann’s equation to calculate the mutual inductance between two current-carrying filaments can be simplified as Equation (2) [31]:

(2) $$M=\rho \times \sum_{i=1}^{i=n_{.Cx}}\sum_{j=1}^{j=n_{.Cy}}M_{ij}\text{ and }M_{ij}=\frac{\mu_{0}\pi a_{i}^{2}b_{j}^{2}}{2{\left(a_{i}^{2}+b_{j}^{2}+d^{2}\right)}^{3/2}}\left(1+\frac{15}{32}\gamma_{ij}^{2}+\frac{315}{1024}\gamma_{ij}^{4}\right)$$

where *a_i_* = *D_out_*_.C*x*_−(*n_._*_C*xi*_1)(*w*_.C*x*_+ *s*_.C*x*_) −*w*_.C*x*_/2, *b_j_* = *D_out._*_C*y*_− (*n_._*_C*yj*_− 1)(*w_._*_C*y*_ + *s_._*_C*y*_) −*w_._*_C*y*_/2, *γ_ij_* = 2*a_i_b_j_*/(*a_i_*^2^ + *b_j_*^2^+ *d*^2^) and *d* is the distance between two adjacent coils. Parameter *ρ* [31] is a structure-dependent term.

Equation (2) estimates all possible *M*s among the planar inductors by considering each turn, and the total *M* can be determined by adding all the combinations. If both TX and RX are circular shaped, *ρ* = 1. Otherwise, for square-shaped TX and RX coils, *ρ* can be approximated as (4/π)^2^. Thus, the *M* value between two square-shaped coils is (4/π)^2^ times greater than that of the circular-shaped coils. 

**Figure 3:**
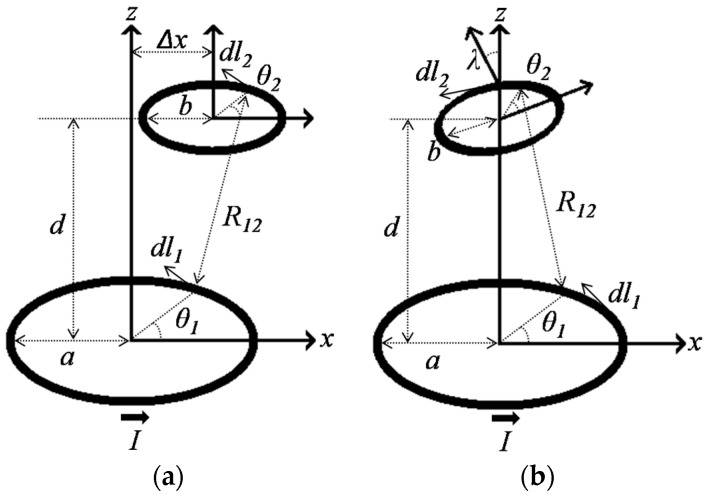
(**a**) Lateral misalignment configuration; (**b**) Angular misalignment configuration.

Figure 3a shows the configuration of the planar coils for lateral misalignment. Δ*x* represents the lateral displacement. 

The distance between two arbitrary points on those coils is assumed as:

$$R_{12}=\sqrt{a^{2}+b^{2}+d^{2}+\Delta x^{2}-2ab\cos(\theta_{1}-\theta_{2})-2\Delta xa\cos\theta_{1}+2\Delta xb\cos\theta_{2}}\;$$

*M* is expressed by using three new parameters [31], where *γ* = 2*ab*/(*a*^2^ + *b*^2^+ *d*^2^+Δ*x^2^*), *α* = 2Δ*xa*/(*a*^2^ + *b*^2^+ *d*^2^+Δ*x^2^*), and *β* = 2Δ*xb*/(*a*^2^ + *b*^2^+ *d*^2^+Δ*x^2^*). Thus:

(3) $$\begin{array}{cc} M= & \frac{\mu_{0}ab}{4\pi {\left(a^{2}+b^{2}+d^{2}+\Delta x^{2}\right)}^{1/2}}\int_{\theta_{2}=0}^{2\pi }\int_{\theta_{1}=0}^{2\pi }\cos(\theta_{1}-\theta_{2})\times \\ & {\left[1-\left(\gamma \cos(\theta_{1}-\theta_{2})+\alpha \cos\theta_{1}-\beta \cos\theta_{2}\right)\right]}^{-1/2}d\theta_{1}d\theta_{2} \end{array}$$

For simplification, the [1 − (*γ*cos(*θ*_1_−*θ*_2_) + *α*cos*θ*_1_−*β*cos*θ*_2_)]^−1/2^ term in Equation (3) can be expanded by Taylor series and solved as:

(4) $$M=\frac{\mu_{0}\pi a^{2}b^{2}}{2{\left(a^{2}+b^{2}+d^{2}+\Delta x^{2}\right)}^{3/2}}[1-\frac{3}{2}\sigma +\frac{15}{32}\gamma^{2}\left(1-\frac{21}{2}\sigma \right)+\frac{15}{16}\left(\alpha^{2}+\beta^{2}\right)\left(1-\frac{7}{4}\sigma \right)]$$

where *σ* = Δ*x^2^*/(*a*^2^ + *b*^2^+ *d*^2^+Δ*x^2^*). 

Figure 3b shows the configuration of angular misalignment and it is represented by *λ*. The distance between two arbitrary points on those coils is evaluated by:

$$R_{12}=\sqrt{a^{2}+b^{2}+d^{2}-2ab\cos\lambda \cos(\theta_{1}-\theta_{2})-2bd\cos\theta_{2}\sin\lambda }\;$$

By introducing the parameter *γ* = 2*ab*cos*λ*/(*a*^2^ + *b*^2^+ *d*^2^) and *α* = 2*bd*sin*λ*/(*a*^2^ + *b*^2^+ *d*^2^), *M* can be expressed as follows:

(5) $$M=\frac{\mu_{0}ab}{4\pi {\left(a^{2}+b^{2}+d^{2}\right)}^{1/2}}\int_{\theta_{2}=0}^{2\pi }\int_{\theta_{1}=0}^{2\pi }\cos(\theta_{1}-\theta_{2})\;\times \;{\left[1-\left(\gamma \cos(\theta_{1}-\theta_{2})+\alpha \cos\theta_{2}\right)\right]}^{-1/2}d\theta_{1}d\theta_{2}$$

*M* can be further simplified by Taylor series from Equation (5) to:

(6) $$M=\frac{\mu_{0}\pi a^{2}b^{2}\cos\lambda }{2{\left(a^{2}+b^{2}+d^{2}\right)}^{3/2}}\left(1+\frac{15}{32}\gamma^{2}+\frac{15}{16}\alpha^{2}\right)$$

To realize a complete planarization of the system, C1 and C2 (as well as C3 and C4) are printed on the same side of the substrate. Therefore, the distance becomes zero between the C1 and C2 coils, along with the C3 and C4 coils. In such condition, Equation (2) reveals a large error in calculating *M*_12_ and *M*_34_. Hence, it can be expanded as Equation (7) [27]:

(7) $$M_{ij}=\frac{\mu_{0}\pi a_{i}^{2}b_{j}^{2}}{2{\left(a_{i}^{2}+b_{j}^{2}+d^{2}\right)}^{3/2}}\left(1+\frac{15}{32}\gamma_{ij}^{2}+\frac{315}{1024}\gamma_{ij}^{4}+...+0.628\gamma_{ij}^{28}\right)$$

### 2.2. Parasitic Capacitance

**Figure 4:**
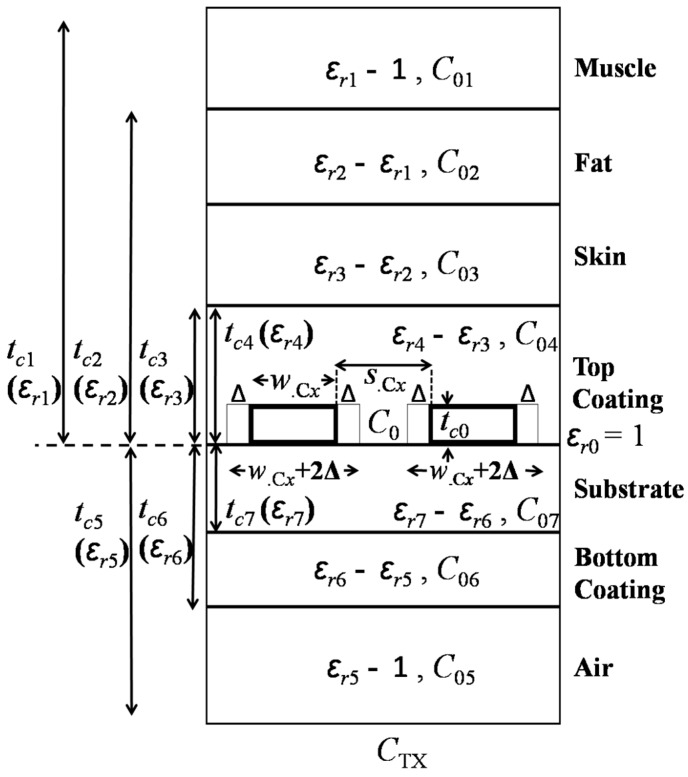
Parasitic capacitance modeling.

Parasitic capacitance, *C_pr_* is a function of the spacing between planar conductive traces and the materials present at the surrounding of the PSCs. High permittivity of the tissue can also increase the parasitic capacitance of the implanted PSC [21]. Figure 4 shows a modeled unit-length parasitic capacitance of a TX coil. 

The PSC is considered as a coplanar stripline sandwiched between multiple dielectric layers. The metal traces of the PSC are printed on a substrate. The top and bottom sides of the substrate are insulated with coating layers. One side of the PSC is human tissue and the other side is air. To realize a more realistic human tissue model, three layers of biological tissues are considered to enhance the approximation accuracy. The general architecture of the biological tissue is formed using consecutive layers of skin, fat and muscle. Moreover, for the RX or implanted coil the air should be replaced by the biological tissue, depending on the anatomical location of the coil. In Figure 4, *t_ci_*, *ɛ_ri_*, *C*_TX_, *C*_0_, and *C*_0*i*_ represent the layer thickness, relative dielectric constant, total capacitance per-unit length of the TX PSC, free-space capacitance of the adjacent traces, and partial capacitance of the dielectric layer, respectively, where, *i* = 0 to 7. According to [32], PSC metal thickness *t_c_*_0_ affects the overall *C*_TX_, and it can be utilized by adjusting the PCS line width and spacing by 2Δ, where:

(8) $$\Delta =\frac{t_{c0}}{2\pi \varepsilon_{a}}\left[1+\ln\left(\frac{8\pi w_{.Cx}}{t_{c0}}\right)\right]$$

(9) $$w_{eff}=w_{.Cx}+2\Delta$$

*ε_a_* represents the mean value of the permittivity of the layers in contact with the PSC strips, and *w_eff_* is the effective width of the PCS metal traces.

An accurate method to approximate *C*_TX_ is presented in [33]. Based on conformal mapping and superposition of the partial capacitances, *C*_TX_ can be simplified as:

(10) $$\begin{array}{cc} C_{\text{TX}} & =\varepsilon_{eff}\times C_{0}\\ & =C_{0}+C_{01}+C_{02}+C_{03}+C_{04}+C_{05}+C_{06}+C_{07} \end{array}$$

where *ε_eff_* is the effective relative dielectric constant.

Theoretically, *C*_0_ can be calculated using Schwartz transformation and can be represented as:

(11) $$C_{0}=\varepsilon_{0}\frac{K^{\prime }(k_{0})}{K(k_{0})},k_{0}=\frac{2s_{.Cx}}{s_{.Cx}+2w_{eff}}$$

where *K* is the complete elliptical integral of the first kind and *K’* (*k_i_*) = *K*(*k_i_’*). *k_i_* and *k_i_’* can be expressed as:

(12) $$k_{i}=\frac{\tanh\left(\frac{\pi s_{.Cx}}{4t_{ci}}\right)}{\tanh\left(\frac{\pi (s_{.Cx}+2w_{.Cx})}{4t_{ci}}\right)}\;\mathrm{and}\;k_{i}^{\prime }=\sqrt{1-k_{i}^{2}}$$

In Figure 4, *ε_eff_* can be obtained from [33]:

(13) $$\begin{array}{cc} \varepsilon_{eff}=1 & +\frac{1}{2}(\varepsilon_{r1}-1)\;\frac{K(k_{0})K(k_{1}^{\prime })}{K(k_{0}^{\prime })K(k_{1})}\;\\ & +\frac{1}{2}(\varepsilon_{r2}-\varepsilon_{r1})\frac{K(k_{0})K(k_{2}^{\prime })}{K(k_{0}^{\prime })K(k_{2})}\\ & +\frac{1}{2}(\varepsilon_{r3}-\varepsilon_{r2})\frac{K(k_{0})K(k_{3}^{\prime })}{K(k_{0}^{\prime })K(k_{3})}\\ & +\frac{1}{2}(\varepsilon_{r4}-\varepsilon_{r3})\frac{K(k_{0})K(k_{4}^{\prime })}{K(k_{0}^{\prime })K(k_{4})}\\ & +\frac{1}{2}(\varepsilon_{r5}-1)\;\frac{K(k_{0})K(k_{5}^{\prime })}{K(k_{0}^{\prime })K(k_{5})}\\ & +\frac{1}{2}(\varepsilon_{r6}-\varepsilon_{r5})\frac{K(k_{0})K(k_{6}^{\prime })}{K(k_{0}^{\prime })K(k_{6})}\\ & +\frac{1}{2}(\varepsilon_{r7}-\varepsilon_{r6})\frac{K(k_{0})K(k_{7}^{\prime })}{K(k_{0}^{\prime })K(k_{7})} \end{array}$$

The ratio of *K*(*k_i_*)/*K’*(*k_i_*) in Equation (13) can be further simplified using the Hilberg approximation and can be expressed as:

(14a) $$\frac{K(k_{i})}{K^{\prime }(k_{i})}\approx \frac{2}{\pi }\ln\left(2\sqrt{\frac{1+k_{i}}{1-k_{i}}}\right),\mathrm{for}\;1\leq \;\frac{K}{K^{\prime }}\leq \infty \;\mathrm{and}\;\frac{1}{\sqrt{2}}\leq k_{i}\leq 1$$

(14b) $$\frac{K(k_{i})}{K^{\prime }(k_{i})}=\frac{\pi }{2\ln\left(2\sqrt{\frac{1+k_{i}^{\prime }}{1-k_{i}^{\prime }}}\right)},\mathrm{for}\;0\leq \;\frac{K}{K^{\prime }}\leq 1\;\mathrm{and}\;0\leq k_{i}\leq \frac{1}{\sqrt{2}}$$

To communicate with the inner terminal, a conductor bridge has to be built. This bridge or via goes across all other turns of the PSC and results in additional parasitic capacitance, which is known as overlapping trace capacitance or *C_tov_*, where:

(15) $$C_{tov}=\varepsilon_{0}\varepsilon_{eff\_ov}\frac{A_{tov}}{t_{tov}}$$

Here, *A*_tov_ is the overlapping area, and *t*_tov_ (≈*t*_c7_) is the spacing between the two metal layers. The effective dielectric constant *ɛ*_eff_ov_ between two conductive plates can be found in [34]:

(16) $$\varepsilon_{eff\_ov}=\frac{\varepsilon_{r7}+1}{2}+\frac{\varepsilon_{r7}-1}{2}{\left(1+\frac{12}{\frac{w_{.Cx}}{t_{c7}}}\right)}^{-\frac{1}{2}}-\frac{\varepsilon_{r7}-1}{4.6}\times \frac{\frac{t_{c0}}{t_{c7}}}{\sqrt{\frac{w_{.Cx}}{t_{c7}}}}$$

Finally, the total parasitic capacitance of the TX PSC can be calculated by:

(17) $$C_{pr}\;=C_{\text{TX}}\cdot l_{c}+C_{tov}.$$

where *l*_c_ is the length of the conductive trace of the PSC for square coil Equation (18a) [22] and circular coil Equation (18b), respectively:

(18a) $$l_{c}=4\cdot n_{.Cx}\;\cdot D_{out.Cx}-4\cdot n_{.Cx}\;\cdot w_{.Cx}-{\left(2n_{.Cx}+1\right)}^{2}(s_{.Cx}+w_{.Cx})$$

(18b) $$l_{c}=2\pi \cdot n_{.Cx}\;\cdot \frac{D_{out.Cx}}{2}-4\cdot n_{.Cx}\;\cdot w_{.Cx}-{\left(2n_{.Cx}+1\right)}^{2}(s_{.Cx}+w_{.Cx})$$

### 2.3. AC Resistance

The Q-factor of the inductor is a function of the effective series resistance (ESR). Thus, to achieve a high Q-factor, the ESR of the inductor coil must be as low as possible. At high frequencies, the skin effect can severely increase. Series resistance *R_s_* is dominated by dc resistance *R_dc_* of the PSC conductive trace:

(19) $$R_{dc}=\rho_{c}\;\frac{l_{c}}{w_{.Cx}\;t_{c0}}$$

where *ρ_c_* represents the resistivity of the PSC conductive material. Skin-effect resistance *R_skin_* can be calculated using Equation (19). Therefore:

(20) $$R_{skin}\;=R_{dc}\cdot \frac{t_{c0}}{\delta \left(1-e^{-\frac{t_{c0}}{\delta }}\right)}\cdot \frac{1}{1+\frac{t_{c0}}{w_{.Cx}}}$$

(21) $$\delta =\sqrt{\frac{\rho_{c}}{\pi \mu f}}\;\mathrm{and}\;\mu =\mu_{0}\cdot \mu_{r}$$

where *δ* is the skin depth, *µ*_0_ is the permeability of free space, *µ_r_* is the relative permeability of the metal layer, and *f* is the operating frequency.

Eddy current is another source of parasitic resistance. The magnetic fields of the external PSC and the adjacent turns of the same PSC can cause eddy current generation. The direction of the eddy currents is opposite that of the main current flow, thus, it increases the PSC effective resistance. The modified resistance by adding the effect of eddy currents [21] can be expressed as:

(22) $$R_{eddy}=\frac{1}{10}R_{dc}{\left(\frac{\omega }{\frac{3.1}{\mu_{0}}\times \frac{s_{.Cx}+w_{.Cx}}{w_{.Cx}^{2}}\times R_{sheet}}\right)}^{2},\;\omega =2\pi f$$

where *R_sheet_* is the metal trace sheet resistance. Consequently, final *R_s_* can be represented as:

(23) $$\begin{array}{l} R_{s}\;=R_{skin}+R_{eddy}\\ =R_{dc}\left(\frac{t_{c0}}{\delta \left(1-e^{-\frac{t_{c0}}{\delta }}\right)}\cdot \frac{1}{1+\frac{t_{c0}}{w_{.Cx}}}+\frac{1}{10}{\left(\frac{\omega }{\frac{3.1}{\mu_{0}}\times \frac{s_{.Cx}+w_{.Cx}}{w_{.Cx}^{2}}\times R_{sheet}}\right)}^{2}\right) \end{array}$$

### 2.4. Q-Factor

**Figure 5:**
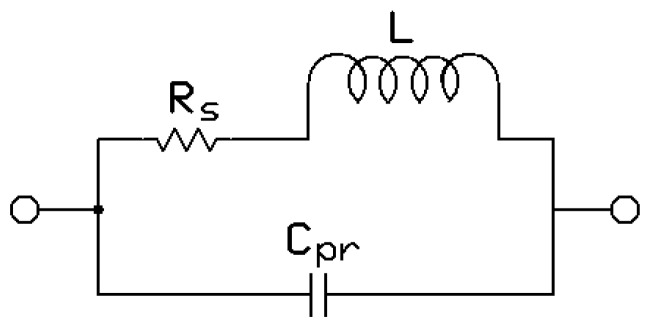
Resonator lumped model.

Figure 5 shows the lumped model of the resonator coil.

Considering that *R_s_* is in series with *L* and *C_pr_* is parallel with both *R_s_* and *L*, the overall impedance of a coil can be expressed as [23]:

(24) $$Z_{e}\;=\left(j\omega L+R_{s}\right)\;\|\;\frac{1}{j\omega C_{pr}}$$

The effective self-inductance *L_eff_* and ESR can be modeled as:

(25) $$\text{ESR}=\frac{R_{s}}{{\left(1-\omega^{2}LC_{pr}\right)}^{2}}$$

(26) $$L_{eff}=\frac{L}{\left(1-\omega^{2}LC_{pr}\right)}$$

The ESR significantly increases as the operating frequency of the coil approaches *f_res_*. For a frequency higher than *f_res_*, the coil behaves as a capacitor, and hence, it cannot be used as an inductor. The Q-factor of an unloaded inductor can be:

(27) $$Q_{unloaded}=\frac{\omega L_{eff}}{ESR}$$

### 2.5. Power Transfer Efficiency

**Figure 6:**
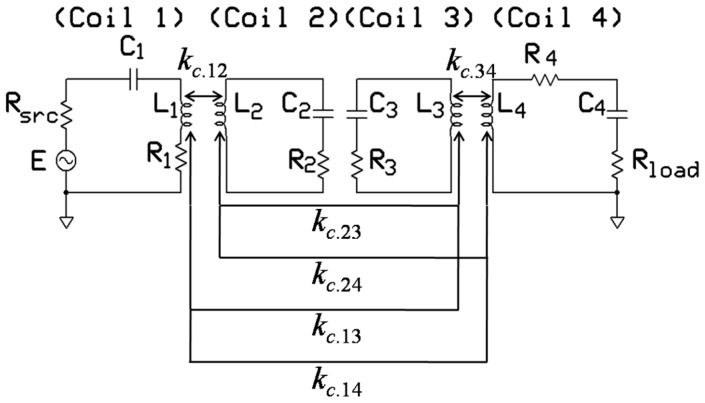
Simplified electric model of the four -coil PTE system.

By applying the circuit theory to Figure 6, the relationship between the current through each coil and the voltage applied to the source can be expressed in the following matrix form [23]:

(28) $$\left[\begin{array}{l} I_{1}\\ I_{2}\\ I_{3}\\ I_{4} \end{array}\right]={\left[\begin{array}{l} Z_{11}\;Z_{12}\;Z_{13}\;Z_{14}\\ Z_{21}\;Z_{22}\;Z_{23}\;Z_{24}\\ Z_{31}\;Z_{32}\;Z_{33}\;Z_{34}\\ Z_{41}\;Z_{42}\;Z_{43}\;Z_{44} \end{array}\right]}^{-1}\;\left[\begin{array}{l} E\\ 0\\ 0\\ 0 \end{array}\right]$$

Equation (28) can be further expanded as [35,36,37]:

(29) $$\left[\begin{array}{l} I_{1}\\ I_{2}\\ I_{3}\\ I_{4} \end{array}\right]={\left[\begin{array}{r} R_{src}+R_{1}+j\omega L_{1}+\frac{1}{j\omega C_{1}}\;j\omega M_{12}\;j\omega M_{13}\;j\omega M_{14}\\ j\omega M_{21}\;R_{2}+j\omega L_{2}+\frac{1}{j\omega C_{2}}\;j\omega M_{23}\;j\omega M_{24}\\ j\omega M_{31}\;j\omega M_{31}\;R_{3}+j\omega L_{3}+\frac{1}{j\omega C_{3}}\;j\omega M_{34}\\ j\omega M_{41}\;j\omega M_{42}\;j\omega M_{43}\;R_{load}+R_{4}+j\omega L_{4}+\frac{1}{j\omega C_{4}} \end{array}\right]}^{-1}\;\left[\begin{array}{l} E\\ 0\\ 0\\ 0 \end{array}\right]$$

where *R_src_* is the source resistance and *R_load_* is the load resistance. *R_src_* and *R*_4_ are small in magnitude, and at resonant frequency, *jωL* = 1/*jωC*. Hence, the imaginary part of the corresponding element is zero. C1 and C4 are small in size; thus, they have a very small inductance and can be neglected. The large distances between C1 and C4, C1 and C3, and C2 and C4 result insignificant mutual inductance and mutual resistance [23,35,38]. Therefore a simplified form of Equation (29) can be expressed as:

(30) $$\left[\begin{array}{l} I_{1}\\ I_{2}\\ I_{3}\\ I_{4} \end{array}\right]={\left[\begin{array}{cccc} R_{1} & j\omega M_{12} & 0 & 0\\ j\omega M_{21} & R_{2} & j\omega M_{23} & 0\\ 0 & j\omega M_{31} & R_{3} & j\omega M_{34}\\ 0 & 0 & j\omega M_{43} & R_{load} \end{array}\right]}^{-1}\;\left[\begin{array}{l} E\\ 0\\ 0\\ 0 \end{array}\right]$$

To simplify PTE equation, *M_ij_* is usually normalized by using *L_i_* and *L_j_* by defining *k_c_*_.*ij*_ as:

(31) $$k_{c.ij}=\frac{M_{ij}}{\sqrt{L_{i}L_{j}}}$$

At resonance, PTE *η* can be expressed as:

(32) $$\;\eta =\frac{\text{Output Power}}{\text{Input Power}}=\;\frac{I_{4}^{2}R_{load}}{I_{1}E}$$

For loaded Q-factor *Q*_4*L*_, Equation (32) can be expanded as [39]:

(33) $$\begin{array}{c} \eta =\eta_{12}\times \eta_{23}\times \eta_{34}\\ \eta =\frac{k_{c.12}^{2}Q_{1}Q_{2}\cdot k_{c.23}^{2}Q_{2}Q_{3}\cdot k_{c.34}^{2}Q_{3}Q_{4L}}{\left[\begin{array}{l} \left[\left(1+k_{c.12}^{2}Q_{1}Q_{2}\right)\cdot \left(1+k_{c.34}^{2}Q_{3}Q_{4L}\right)+k_{c.23}^{2}Q_{2}Q_{3}\right]\times \\ \left(1+k_{c.23}^{2}Q_{2}Q_{3}+k_{c.34}^{2}Q_{3}Q_{4L}\right) \end{array}\right]}\times \frac{Q_{4L}}{Q_{L}} \end{array}$$

where *Q_L_* (=*R_load_*/*ωL*_4_) is the load Q-factor and *Q*_4*L*_ = (*Q*_4_·*Q_L_*)/(*Q*_4_ + *Q_L_*). The PTE (*η*_23_) of loosely coupled C2 and C3 is the dominant factor in determining the overall PTE of the four-coil link at large coupling distance *d*. In Equation (27), the effects of adjacent coils in a multi-coil-based system are ignored. In contrast, Q-factor can be estimated more accurately by considering the effect of reflected impedance from the load coil back to the driver coil, one stage at time in a multi-coil system. At resonance, the effect of RX on TX can be modeled using the reflected impedance [40]:

(34) $$R_{ref.j,j+1}=k_{c.j,j+1}^{2}\;\omega L_{j}Q_{(j+1)L},j=1,2,...\;m-1$$

In Equation (34), *R_ref.j,j+1_* represents the reflected load from (*j* + 1)th to *j*th coil, where *k_c.j,j+1_* is the coupling coefficient between the *j*th and (*j* + 1)th coils and all coils are tuned at *f_res_*. *Q_(j+1)L_* is the loaded Q-factor of the (*j* + 1)th coil, which can be obtained from:

(35) $$Q_{jL}=\frac{\omega L_{j}}{R_{j}+R_{ref.j,(j+1)}}\;=\frac{\frac{\omega L_{j}}{R_{j}}}{1+k_{c.j,j+1}^{2}\;\left(\frac{\omega L_{j}}{R_{j}}\right)Q_{(j+1)L}}\;=\frac{Q_{j}}{1+k_{c.j,j+1}^{2}Q_{j}Q_{(j+1)L}}\;$$

where, *Q_j_* = *Q_unloaded_* = (ω*L_j_*/*R_j_*) and *R_j_* are the unloaded quality factor Equation (27) and parasitic series resistance of the *j*th coil, respectively. According to Equation (34), when *R_Load_* is reflected onto C3 through C4, it limits the Q-factor of C3 [40]. Similarly, the total impedance in the secondary coil (C3) is reflected onto the primary coil (C2) and reduces the Q-factor of C2 (*Q*_2_ = *ωL*_2_/*R*_2_), which can be expressed as:

(36) $$Q_{2L}=\frac{Q_{2}}{1+k_{c.23}^{2}Q_{2}Q_{3L}}\;$$

From Equation (36) it can be inferred that strong coupling between C2 and C3 (i.e., high *k_c._*_23_) reduces *Q_2L_* and thus, *η*_12_, which is the PTE between C1 and C2. In Equation (36) *Q*_2*L*_ is roughly proportional to *k_c._*_23_^−2^, where *k_c._*_23_ is further inversely proportional to *d*^2^ [23]. Therefore, *Q*_2*L*_ is proportional to *d*^4^. For a small *d*, *Q*_2*L*_ will reduce significantly. It implies that *η*_12_ will reduce enormously as well. *η*_12_ can be expressed as [40]:

(37) $$\eta_{12}=\frac{k_{c.12}^{2}Q_{1}Q_{2L}}{1+k_{c.12}^{2}Q_{1}Q_{2L}}\times \frac{Q_{2L}}{Q_{L}}\;$$

In Equation (37), *η*_12_ is significantly reduced at small *d* if *k_c._*_12_ is not chosen to be large. According to Equation (34), large *k_c._*_12_ results in a large reflected load on C1, which can reduce the available power from the source. On the other hand, PDL *P_load_* can be calculated by multiplying the power provided by power source *E* by the PTE as [40]:

(38) $$P_{load}=\frac{E^{2}}{2R_{ref.1,2}}\times \eta$$

## 3. Design and Optimization Procedure

In this section, the design steps for a fully planar four-coil MRC WPT are presented using the theoretical models of Section 2. These steps are presented in the context of optimizing the design constraints and achieving maximum PTE with an utilizable PDL for implantable device. A MATLAB model of Equations (1)~(33) is developed and used to optimize the architecture of the coils to achieve high PTE. The parasitic modeling are also utilized to engender appropriate optimization. The high-frequency structure simulator ANSYS-HFSS-15.0 with circuit modeling and the simulation software ANSYS-Simplorer is used to simulate and validate the optimization of the size of the TX and RX coils. 

### 3.1. Design Constraints

The first step is to specify the design parameters of the four-coil WPT system for biomedical implants. Table 1 lists the design constraints in terms of the size, coupling distance, fabrication technology, carrier frequency, and load resistance.

### 3.2. Parameter Initialization

**Figure 7:**
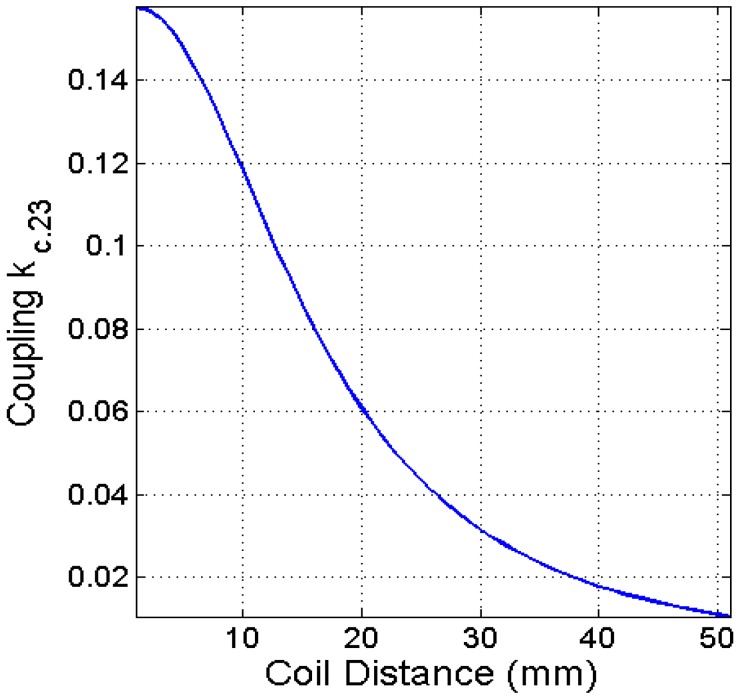
Mutual coupling (*k_c._*_23_) versus distance.

Before the iterative optimization process is started, a set of values are needed to be initialized. To realize a high PTE in a relatively compact structure, the design initialization starts with the coupling coefficient (*k_c._*_23_) [23,41]. It is a generalized approximation for both square and circular coils:

(39) $$k_{c.23}\;=148.2{\left(\frac{1}{d^{2}+r_{m}^{2}}\right)}^{1.2}-0.0002857$$

where *r_m_* is the geometric mean of the radius of the TX and RX coils. From Equation (39) and Table 1, the estimated *k_c._*_23_ is 0.1182 when *d* = 10 mm, which is shown in Figure 7.

**Figure 8:**
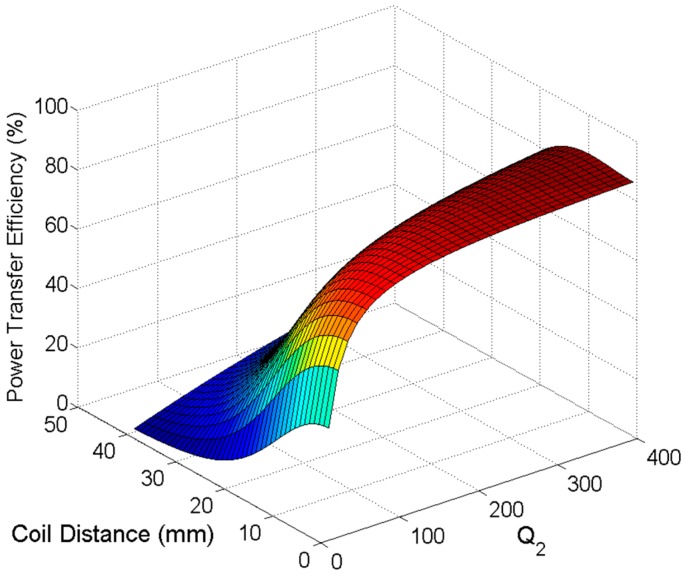
Efficiency versus Q-factor versus distance.

The maximum achievable PTE in Equation (33) at a 10-mm relative distance between the TX and RX coils is 84% for the specified *k_c._*_23_ value. Figure 8 shows that the maximum PTE is achieved for C2 Q-factor *Q_2_* = 226. The minimum necessary Q-factor for C1 and C4 can be approximated from $Q_{1,4}>Q_{2,3}^{-0.5}$ . The coupling coefficients and Q-factors of C1, C3, and C4 in Equation (33) are initialized, as listed in Table 2. It lists the parameters [22,23,27] that are iteratively optimized by using MATLAB in the further parts of this paper to achieve maximum PTE. Simultaneously, the optimized architecture is validated using HFSS. Based on the initial values of Table 2 the TX and RX coils are modeled in HFSS. In order to reduce the power losses in transmission load, copper material with 0.038 mm thickness is printed on FR4-epoxy substrate with constitutive parameters of 1.6 mm substrate thickness, 4.4 relative permittivity (*ɛ_r_*), 0.02 dielectric loss tangent, and “1” relative permeability (*µ*_r_). Resonant circuit similar to Figure 6 is modeled and simulated in Simplorer to measure the PTE of the HFSS modeled coils at the operating frequency of 13.56 MHz.

### 3.3. Size and Number of Turns of Primary PSC

To optimize the size of the primary PSC, the values approximated in steps 1 and 2 are used in Equations (1)~(33). By keeping *s* and *w* constant, *D_out_*_.C2_ and *n*_.C2_ are swept in a wide range around their initial values to extract the highest PTE. According to [22], *n*_.C2_ can be expressed as a function of *ϕ*_2_:

(40) $$n_{.C2}=\frac{D_{out.C2}}{s_{.C2}+w_{.C2}}\cdot \frac{\varphi_{2}}{\left(1+\varphi_{2}\right)}$$

**Figure 9:**
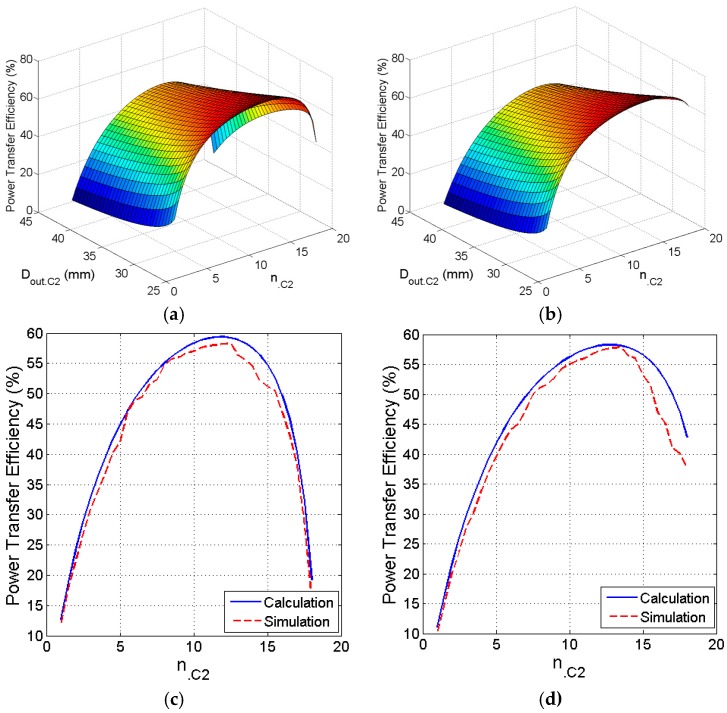
Optimization of the size and number of turns of the primary PSC. (**a**) Efficiency versus outer diameter versus number of turns of the square coil; (**b**) Efficiency versus outer diameter versus number of turns of the circular coil; (**c**) Calculated and simulated efficiency versus number of turns of the square coil; (**d**) Calculated and simulated efficiency versus number of turns of the circular coil.

For a square-shaped coil, Figure 9a shows that the best choice for *D_out_*_.C2_ is 40 mm. The maximum PTE is 59.66% when the number of turns *n*._C2_ = 12. The PTE should improve once *w*_.C2_ and the size of the secondary PSC are optimized. Similarly, the circular coil shown in Figure 9b reaches its peak *η* = 57.84% at *D_out_*_.C2_ = 38.5 mm and *n*._C2_ = 11.5. Figure 9c shows calculated and simulated values of PTE versus *n*._C2_ at *D_out_*_.C2_ = 40 mm for square coil. Similarly, Figure 9d shows calculated and simulated values of PTE versus *n*._C2_ at *D_out_*_.C2_ = 38.5 mm for circular coil.

### 3.4. Fill Factor and Line Width of Secondary PSC

**Figure 10:**
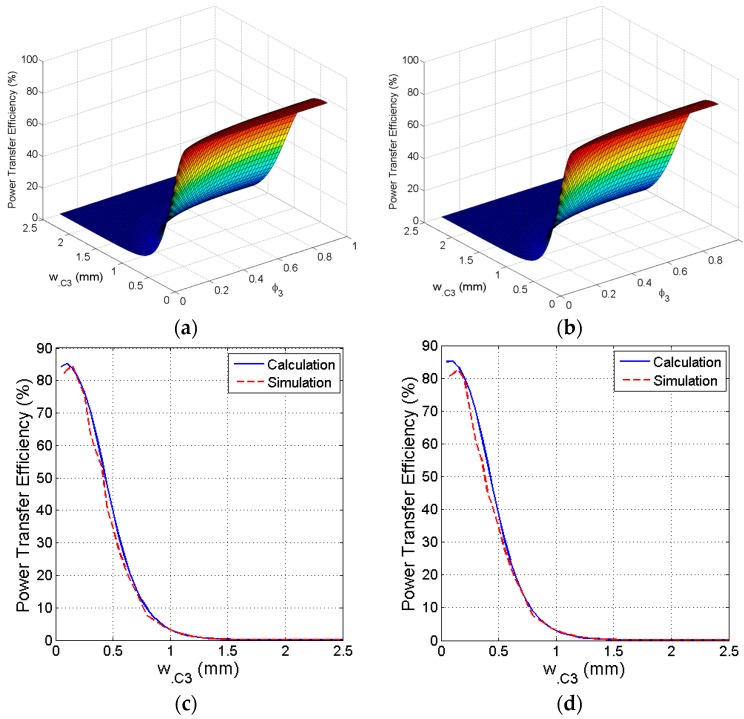
Optimization of the trace line width and fill factor of the secondary PSC. (**a**) Efficiency versus line width versus fill factor of the square coil; (**b**) Efficiency versus line width versus fill factor of the circular coil; (**c**) Calculated and simulated efficiency versus line width of the square coil; (**d**) Calculated and simulated efficiency versus line width of the circular coil.

After temporarily fixing the parameters of the primary PSC in the previous step, the next step generalizes the structure parameters of the secondary coil. Considering that *D_out_*_.C3_ is predetermined in the design constraints, in this step, *ϕ*_3_ is swept around its nominal value and increases *w*_.C3_ from its minimum value. Figure 10a shows that the maximum PTE of a square PSC is slightly below the specified minimum value of *w*_.C3_. Thus, the selected value of the secondary PSC line width is *w*_.C3_ = *w*_.C3__(minimum)_. According to the initialized value, *D_in_*_.C3_ = 12.3 mm. Therefore, to manage the size constraints and maximum PTE trade-off, we select *ϕ*_3_ = 0.24, which corresponds to *n*._C3_ = 12.9 and yields *η* = 83%.

Similar to the square secondary PSC parameters, Figure 10b shows the approximation of the parameters of the circular secondary PSC. Maximum PTE *η* = 82.19% can be achieved for *w*_.C3_ = *w*_.C3(minimum)_ by considering the size constraints as well as the initial values. To obtain the peak PTE we select *ϕ*_3_ = 0.26, which yields *n*._C3_ = 13.75. Figure 10c shows calculated and simulated values of PTE versus *w*_.C3_ at *ϕ*_3_ = 0.24 for the square coil. Similarly, Figure 10d shows calculated and simulated values of PTE versus *w*_.C3_ at *ϕ*_3_ = 0.26 for the circular coil.

### 3.5. Size and Line Width of Primary PSC

**Figure 11:**
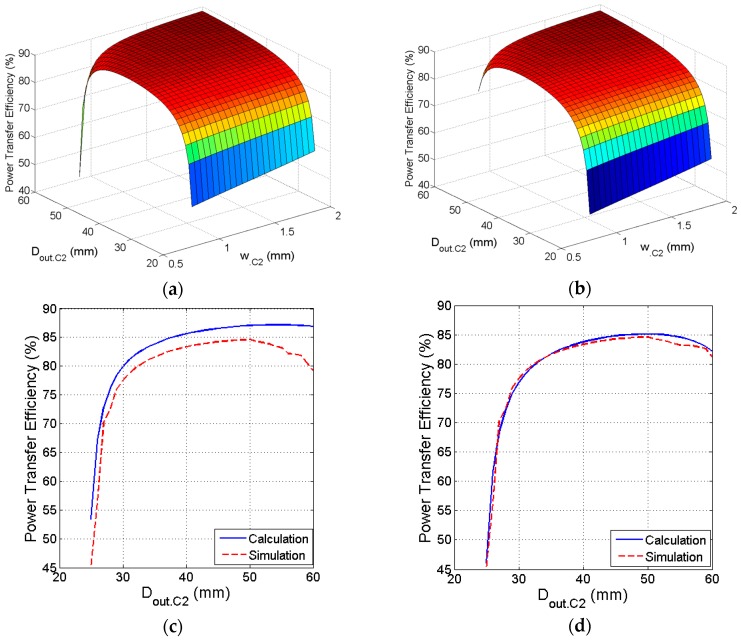
Optimization of the trace line width and size of the primary PSC. (**a**) Efficiency versus line width versus outer diameter of the square coil; (**b**) Efficiency versus line width versus outer diameter of the circular coil; (**c**) Calculated and simulated efficiency versus outer diameter of the square coil; (**d**) Calculated and simulated efficiency versus outer diameter of the circular coil.

In this step, the conductor width *w*_.C2_ is increased toward its optimum value while the outer diameter is also increased to provide room for the excess *w*_.C2_. The increase in *w*_.C2_ reduces *R_s_*_.C2_ and increases *Q*_2_. Hence, the overall PTE is improved. Figure 11a shows that the PTE reaches its peak *η* = 86.85% for optimal value of *w*_.C2_ = 1.2 mm and *D_out_*_.C2_ = 57 mm by considering *n*._C2_ = 12, which is obtained from the previous step. Following the same steps for the circular PSC, Figure 11b shows the peak PTE at *η* = 85.01% for *w*_.C2_ = 1.05 mm and *D_out_*_.C2_ = 52 mm. The number of turns is fixed at *n*._C2_ = 11.5, which is extracted from the previous step. Figure 11c shows calculated and simulated values of PTE versus *D_out_*_.C2_ at *w*_.C2_ = 1.2 mm for the square coil. Similarly, Figure 11d shows calculated and simulated values of PTE versus *D_out_*_.C2_ at *w*_.C2_ = 1.05 mm for the circular coil.

### 3.6. Iteration and Validation

**Figure 12:**
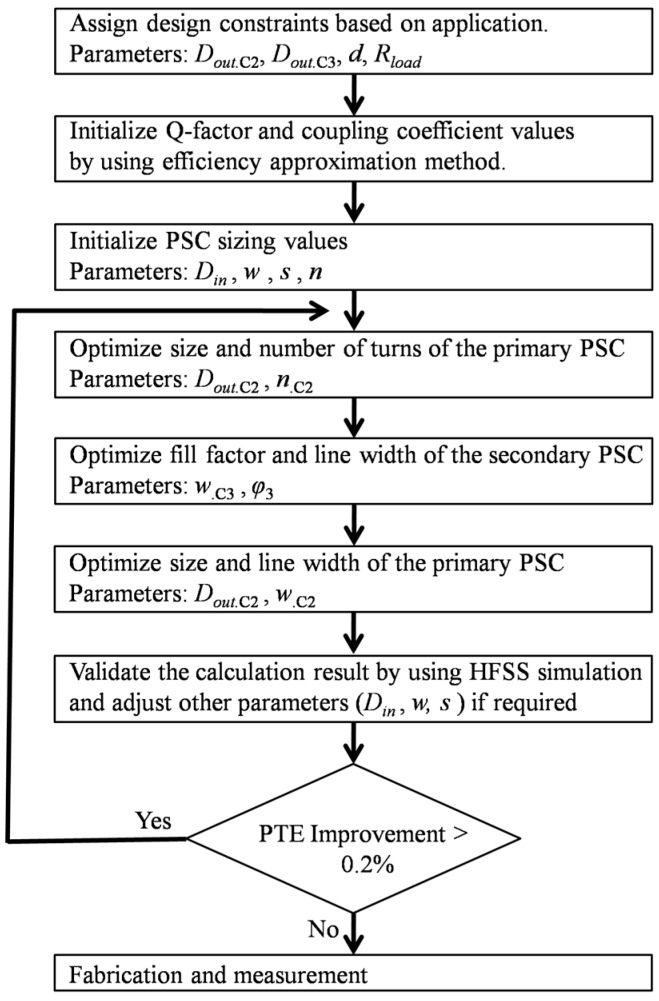
Iterative design flowchart.

The previous step shows that by updating the geometric size, the PTE can be significantly improved. On the other hand, further improvement is possible by iterating the whole optimization procedure. To achieve higher efficiency, some parameters (*D_in._*_C1_, *D_in._*_C4_, *w_._*_C1_, *w_._*_C4_, and *s*) are also adjusted during simulation in HFSS. The iteration process can be continued until the improvement in PTE per iteration is less than 0.2%. Table 3 lists the final optimized geometric values, and Figure 12 shows the summary of the iterative PSC design procedures in a flowchart form.

## 4. Fabrication and Results

**Figure 13:**
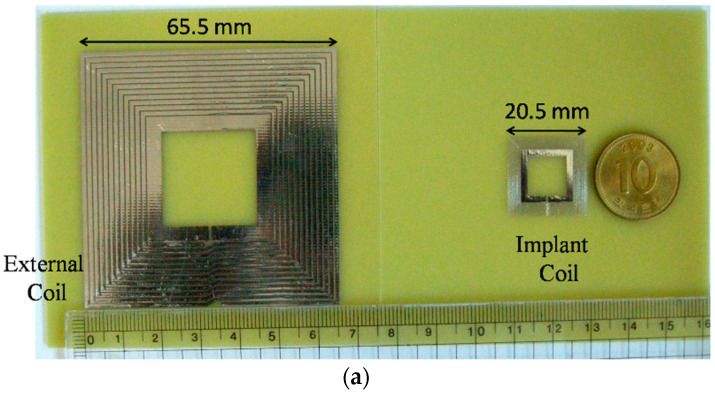

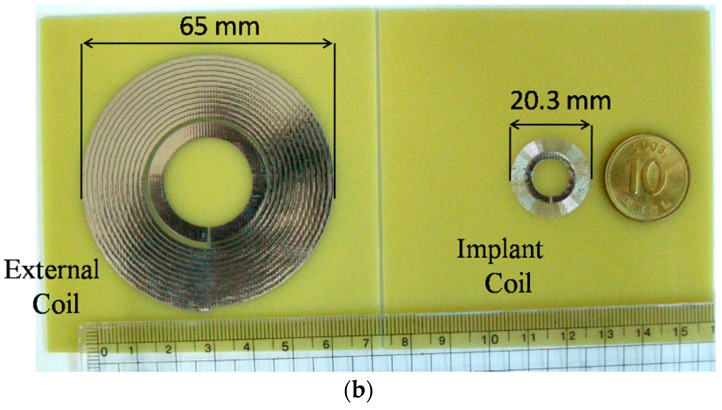
Coil dimension. (**a**) Square coil. (**b**) Circular coil.

To verify the accuracy of the PTE and PDL equations through measurement, both the square and circular fully planar four-coil MRC-WPTs are fabricated on 1.6-mm-thick FR4 printed circuit boards. Figure 13a,b shows the fabricated square and circular coils, respectively. For a nominal coupling distance of *d* = 10 mm, *R*_load_ = 100 Ω, and *f* = 13.56 MHz, Table 4 lists the electrical specifications of the designed coils achieved from simulation.

**Figure 14:**
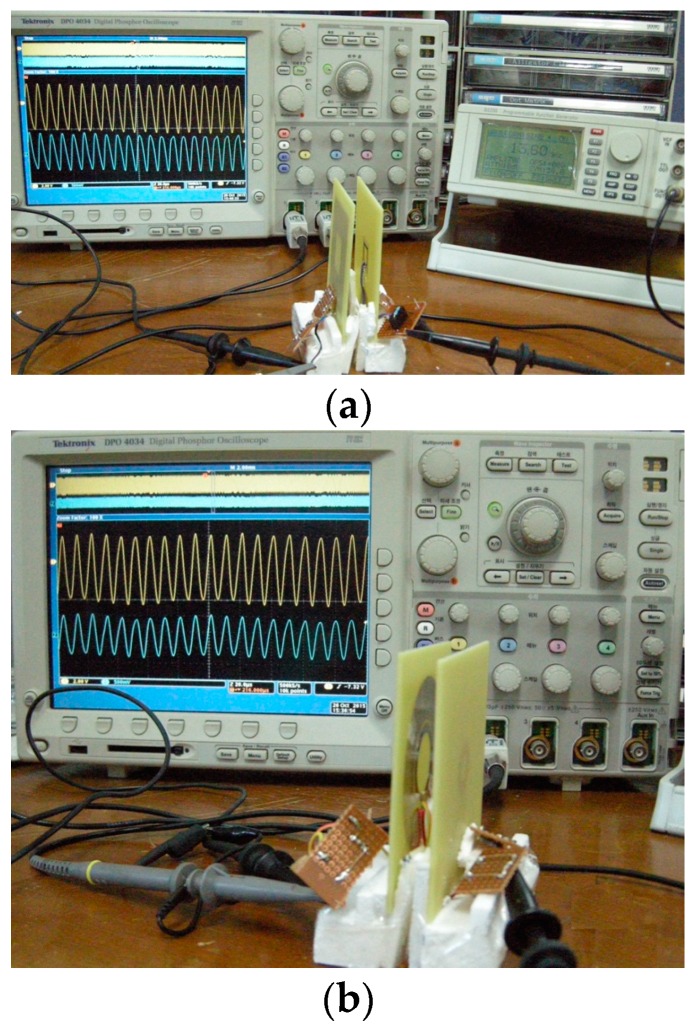
Experimental setup. (**a**) Square coil; (**b**) Circular coil.

Figure 14 shows the experimental setup [35] to verify the fabricated coils. Resonant circuit similar to Figure 6 is built for both the square and circular coils. A signal generator is used as a power source and *R*_src_ = 50 Ω. For the measurement, an oscilloscope (DPO 4034, Tektronics, Beaverton, OR, USA) and an LCR meter are used. The transmitted and received power can be easily determined by measuring the current and voltage waveforms.

**Figure 15:**
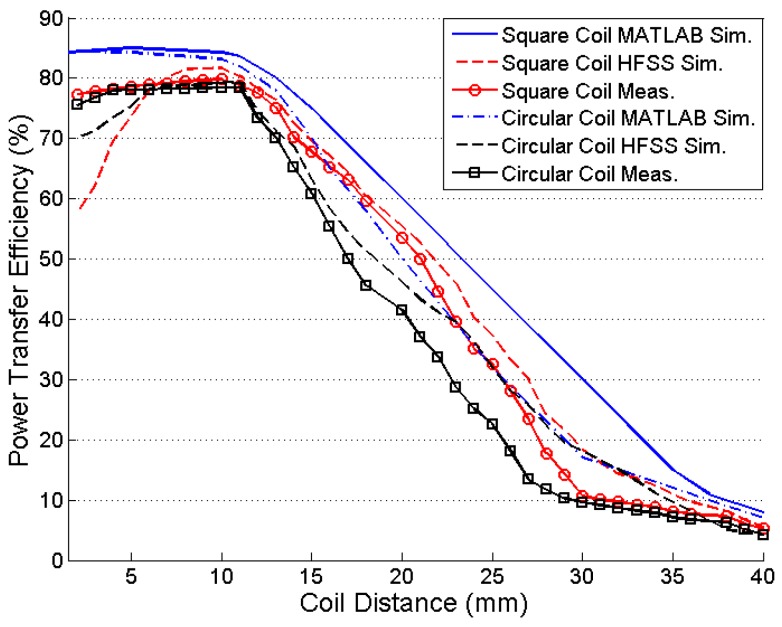
Efficiency versus coupling distance.

Figure 15 shows the comparison of the calculated, simulated, and measured values of the PTE versus coupling distance in the square and circular coil inductive links. The designed coils are optimized for 10 mm of coupling distance and generate a maximum PTE at that specified coupling distance. High *R*_src_ is an important factor to degrade the PTE performance from the MATLAB simulation to HFSS simulation and measurement data. During MATLAB simulation of PTE in Equation (33) the effect of *k_c._*_14_, *k_c._*_24_, and *k_c._*_13_ are neglected. At *d* < 5 mm, these parameters cannot be neglected. The effect of *R*_src_ in Equation (30) is not considered as well for the simplicity of the calculation and simulation in MATLAB. Thus, there is a discrepancy visible between the calculation (MATLAB simulation) and measured data (HFSS simulation and measured) at *d* < 5 mm. 

**Figure 16:**
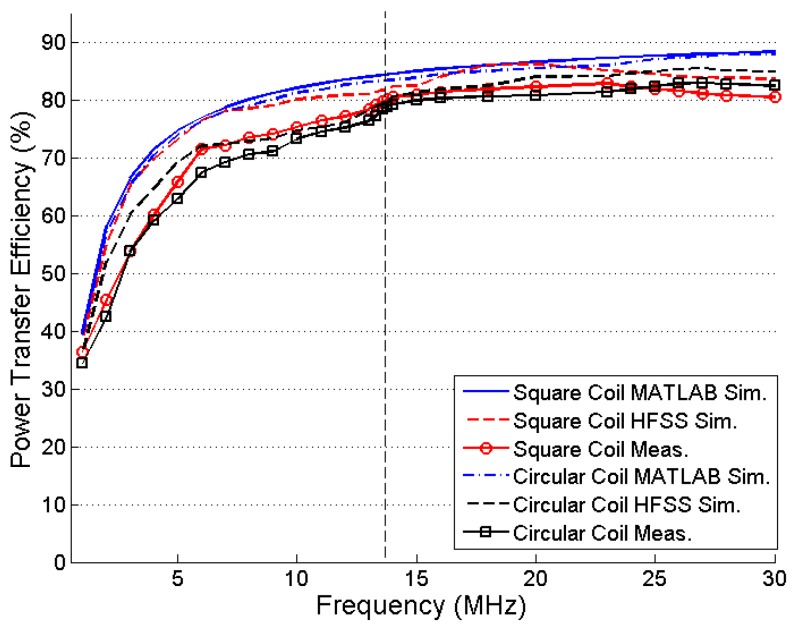
Efficiency versus carrier frequency.

For a frequency sweep of 500 Hz to 30 MHz, the PTE is maximized at approximately 13–14 MHz because of the high Q-factor of the PSCs for both the square and circular coils, as shown in Figure 16. The improvement in PTE is very small at higher frequencies. 

**Figure 17:**
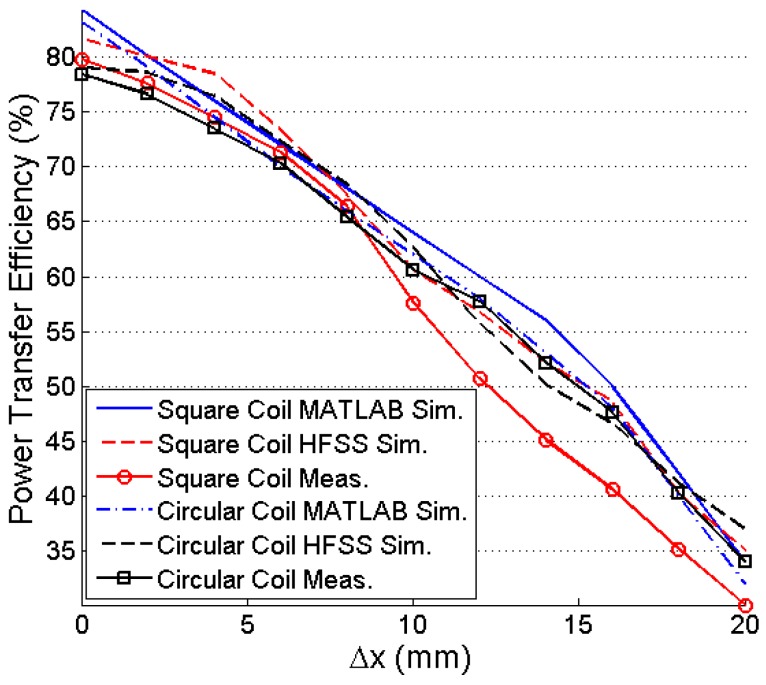
Efficiency versus lateral misalignment.

**Figure 18:**
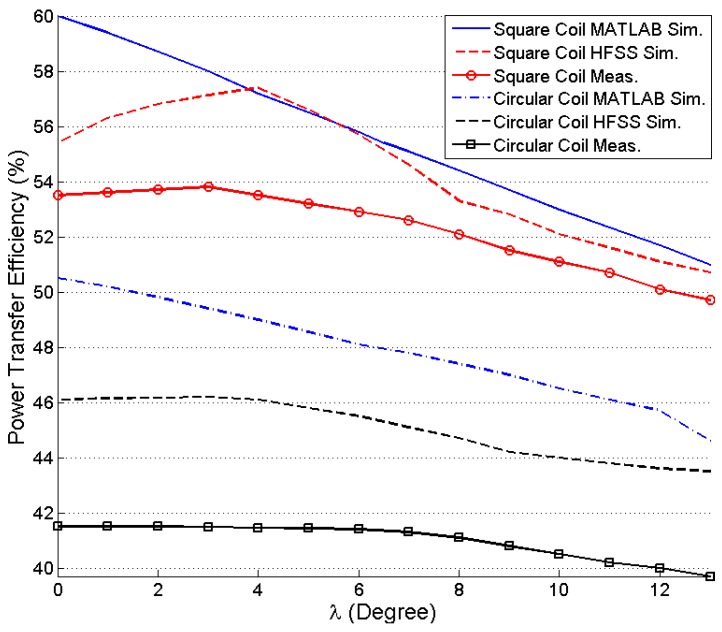
Efficiency versus angular misalignment (*d* = 20 mm).

Figure 17 shows the effect of lateral misalignment on PTE. The variation of PTE for Equation (4) is simulated using MATLAB and verified by HFSS simulation and measurement. TX coil is kept constant and RX coil’s positions are changed from 0 mm to 20 mm lateral plane for both simulation and experimental measurement at *d* = 10 mm. The PTE decreases significantly for higher lateral displacement of the RX coil. The effect of angular misalignment on PTE is observed for MATLAB simulation, HFSS simulation, and practical measurement on Figure 18. For *d* = 20 mm the PTE is simulated and measured for *λ* = 0 to 13 degree. The PTE decreases exponentially in both the cases of lateral and angular misalignments.

**Figure 19:**
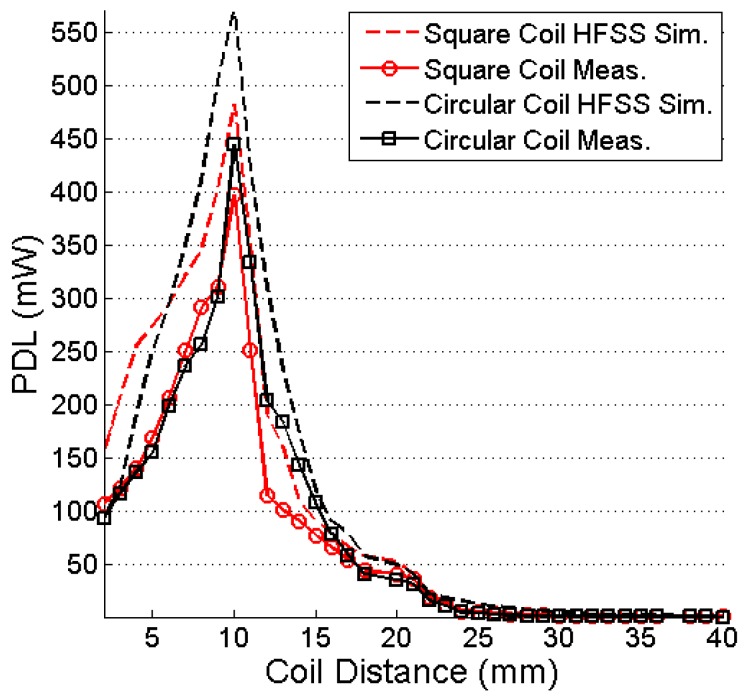
PDL versus coupling distance.

**Figure 20:**
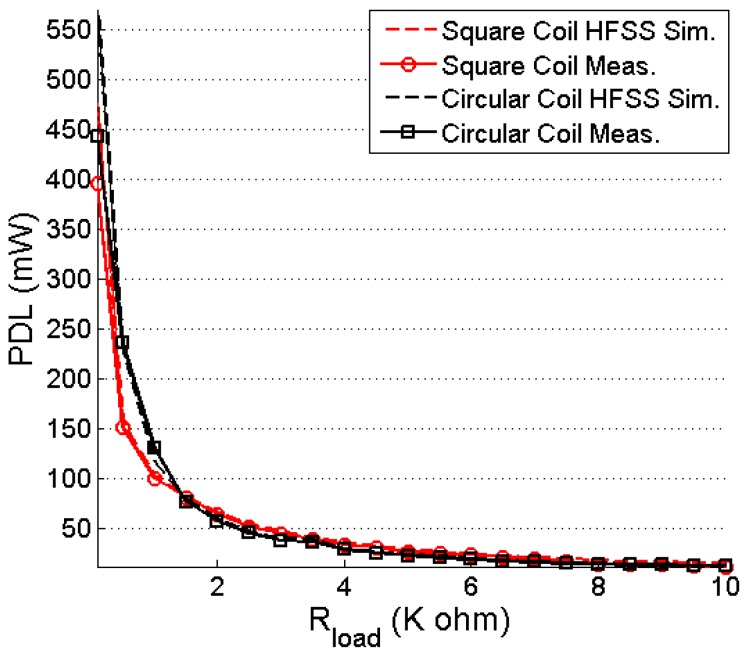
PDL versus R_load_.

The output power at a 100-Ω load resistance of the system is simulated and measured, and its plot is shown in Figure 19. For 10 mm of coupling distance between the TX and RX coils and 13.56-MHz frequency, the maximum simulated and measured PDLs for the square coil resonator are 481.76 and 396 mW, respectively. Table 4 lists the comparison of the coupling coefficients between the square and circular coils. Thus, Equation (37) justifies the higher PDLs of the circular coil, which are 570.35 and 443.5 mW, respectively, under the simulation and measurement conditions. Figure 20 shows the effect of changing load on the received power. The PDL is reduced significantly for a 10 K-Ω load resistor. The minimum simulated and measured received power is 14.6 mW and 11.7 mW, respectively, for the square coil resonator for 10 K-Ω load resistor. In case of circular coil, simulated and measured PDLs are 11.85 mW and 12.85 mW, respectively, for a 10 K-Ω load.

Table 5 lists the summary and comparison of the work presented in this paper with previous works. The comparison is focused only on the four-coil based resonators for different applications where the coupling medium and surrounding environment is air. Both the square and circular coils are also simulated in a biological tissue medium. In contrast to the air medium, the PTE significantly degrades in the tissue medium. In HFSS, the 10-mm tissue medium is created similar to that shown in Figure 1, where we consider that the skin, fat, and muscle-tissue thicknesses are 1, 2, and 7 mm, respectively. Table 6 lists the electrical properties of the mentioned human tissues.

**Figure 21:**
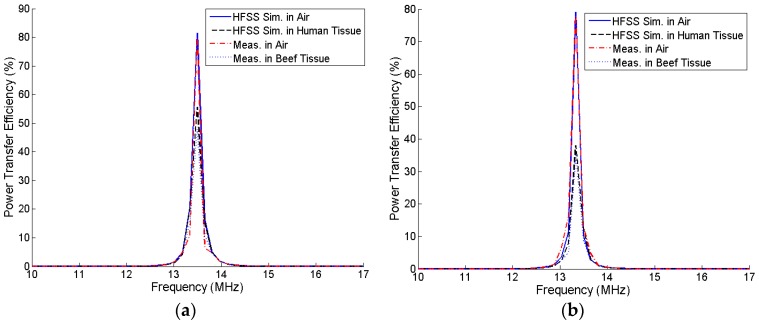
Comparison of the simulated PTE between the air and three-layered human-tissue media at resonance. (**a**) Square coil; (**b**) Circular coil.

**Figure 22:**
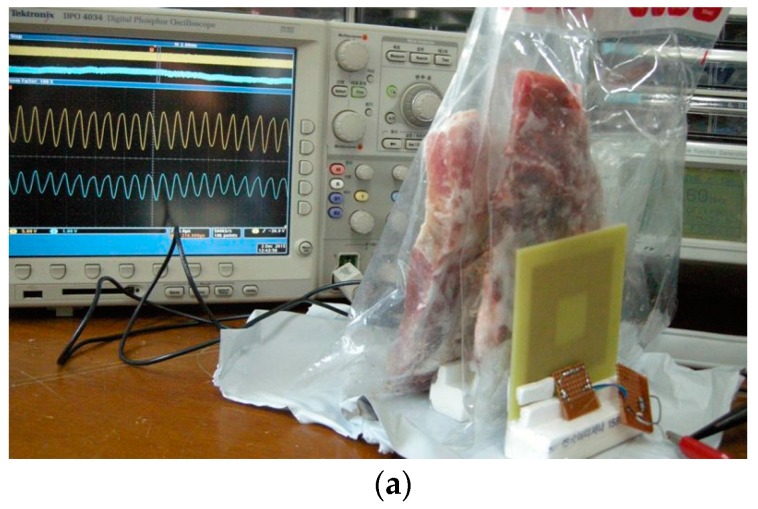

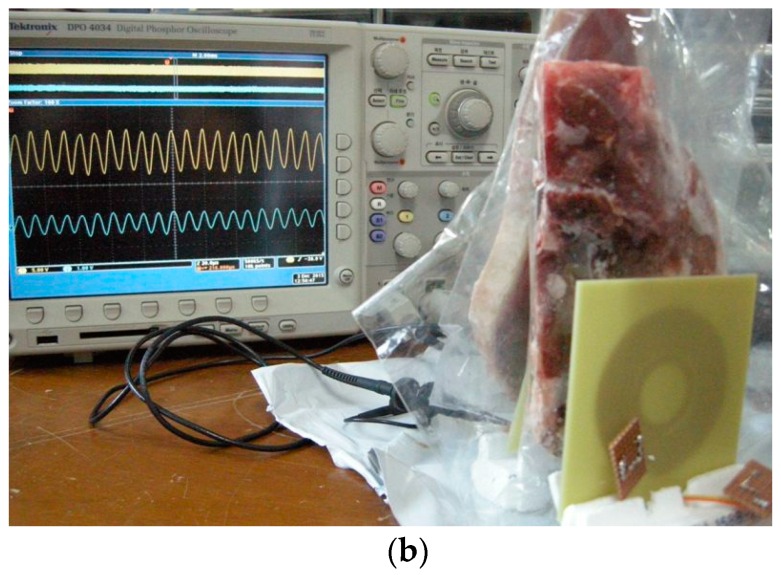
Experimental setup of the PTE measurement in the beef muscle tissue medium. (**a**) Square coil; (**b**) Circular coil.

Figure 21 shows the comparison of the simulated and measured PTE of the square and circular PSCs in air and human tissue media under resonance condition. Instead of real human tissue, beef tissue medium was used for the practical measurements. The simulated PTEs of the square and circular coils are degraded to 55.74% and 38.06%, respectively, when the coupling medium between the TX and RX coils is a three-layered human tissue. Figure 22 shows the experimental setup to measure the PTE when the coupling medium between the TX and RX coils is 10 mm of beef muscle tissue, which are cut from the lower portion of the ribs. A 20-mm of beef muscle tissue layer is also placed behind the RX coil. The temperature of the beef muscle was 8.7 °C at the time of measurement, and the measured PTE is 48.1% and 35.4%, respectively, for the square and circular PSCs. Due to high permittivity and high conductivity of the biological tissue environment, the Q-factor of each PSC drops significantly. It also affects the parasitic parameters of the PSC. Thus, the PTE is decreased drastically in the biological tissue medium than the air medium.

## 5. Conclusions

In this paper, a detailed analysis of a fully planar MRC-WPT system for a four-coil-based architecture has been presented for both square and circular structures. The proposed models are optimized using an iterative procedure and fabricated to validate the theoretical modeling. From the analysis, we determined that the circular PSCs require lower self- and mutual inductances than the square PSCs and as a result, the Q-factor and PTE of a circular resonator can be less than those of a square resonator. Another assumption was that a low coupling coefficient can cause a low PTE, but a high PDL due to lower reflection at the load. This phenomenon is also verified. The coupling coefficient of the circular PSC is lower than that of square PSC. Hence, circular PSC provides higher PDL than the square PSC. The designed and fabricated structures are also verified in a biological tissue medium. Very few previous attempts considered the tissue medium. In case of biomedical applications, only an air-medium reference cannot offer an appropriate PTE scenario. On the other hand, most of the previous works overlooked the PDL and only dealt with the PTE performance. Even high- PTE systems can transfer a low PDL. In this work, the PDL for both coil configurations (square and circular) are measured and compared. Although the biological tissue medium reduces the PTE, the amount of power that can be received by the designed square or circular implanted coil remains sufficient to drive a sub-micron technology-based bio-implantable device. Misalignment between external and internal coils is very important issue in a transcutaneous system. Thus, the effect of misalignment on PTE is characterized and practically observed. Compactness of the implanted coil is a major requirement for biomedical applications. A fully planar architecture is more suitable than a simple planar or Litz coil- based resonators in this manner. Thus, the proposed four-coil fully planar MRC-WPT can be a good candidate for biomedical devices.

## Figures and Tables

**Table 1:** Design Constraints.

Parameters	Symbol	Design Value
TX outer diameter	*D_out._*_C2_	≤60 mm
RX outer diameter	*D_out._*_C3_	≤20 mm
Coil thickness	*t_c0_*	38 µm
Conductor material properties	*ρ_c_, µ_r_*	~17 nΩm, ~1
Substrate thickness	*t_c7_*	1.6 mm
Substrate dielectric constant	*ɛ_r7_*	4.4 (FR4)
Link operating frequency	*f_res_*	13.56 MHz
Coil relative thickness	*d*	10 mm
Load resistance	*R_load_*	100 Ω

**Table 2:** Initial Values.

Parameters	Symbol	Value
C1 inner diameter	*D_in._*_C1_	20 mm
C4 inner diameter	*D_in._*_C4_	8 mm
C1 line width	*w_._*_C1_	2 mm
C1 line spacing	*s_._*_C1_	150 µm
C2 line width	*w_._*_C2 (minimum)_	150 µm
C2 line spacing	*s_._*_C2 (minimum)_	150 µm
C3 line width	*w_._*_C3 (minimum)_	150 µm
C3 line spacing	*s_._*_C3 (minimum)_	150 µm
C4 line width	*w_._*_C4_	2 mm
C4 line spacing	*s_._*_C4_	150 µm
C1 number of turns	*n_._*_C1_	1
C4 number of turns	*n_._*_C4_	1
C1 Q-factor	*Q*_1_	4
C3 Q-factor	*Q*_3_	120
C4 Q-factor	*Q*_4_	1.4
C1 & C2 coupling coefficient	*k_c._*_12_	0.5
C3 & C4 coupling coefficient	*k_c._*_34_	0.45

**Table 3:** Optimized Geometric Values.

Parameters	Square Coil	Circular Coil	Parameters	Square Coil	Circular Coil
*D_in._*_C1_ (mm)	24	24	*w_._*_C2_ (mm)	1.24	1.21
*D_out._*_C1_ (mm)	32	32	*s_._*_C2_ (µm)	150	150
*D_in._*_C2_ (mm)	32.3	32.6	*w_._*_C3_ (µm)	170	150
*D_out._*_C2_ (mm)	65.5	65	*s_._*_C3_ (µm)	150	150
*D_in._*_C3_ (mm)	13.9	14	*w_._*_C4_ (mm)	1.8	1.8
*D_out._*_C3_ (mm)	20.5	20.3	*s_._*_C4_ (µm)	150	200
*D_in._*_C4_ (mm)	10	10	*n_._*_C1_	1	1
*D_out._*_C4_ (mm)	13.6	13.6	*n_._*_C2_	12	12
*w_._*_C1_ (mm)	4	4	*n_._*_C3_	10	10
*s_._*_C1_ (µm)	150	300	*n_._*_C4_	1	1

**Table 4:** Electrical Specification.

Parameters	Square Coil	Circular Coil	Parameters	Square Coil	Circular Coil
*L*_1_ (nH)	78.78	67.6	*Q*_1_	2.55	2.44
*L*_2_ (µH)	9.25	8.03	*Q*_2_	313.6	302.7
*L*_3_ (µH)	3.09	2.59	*Q*_3_	97.76	92.32
*L*_4_ (nH)	44.07	50.1	*Q*_4_ (loaded)	0.84	0.82
*C*_1_ (nF)	1.76	2.04	*k_c._*_12_	0.32	0.287
*C*_2_ (pF)	14.9	17.16	*k_c._*_23_	0.112	0.11
*C*_3_ (pF)	44.5	53.2	*k_c._*_34_	0.33	0.27
*C*_4_ (nF)	3.13	2.75	*M*_23_ (nH)	594.6	504.06
*R*_1_ (Ω)	0.05	0.047	*d* (mm)	10	10
*R*_2_ (Ω)	2.51	2.26	*η* (simulated)	81.66%	79.07%
*R*_3_ (Ω)	2.82	2.39	*η* (measured)	79.8%	78.43%
*R*_4_ (Ω)	0.04	0.052			

**Table 5:** Comparison with Previous Work.

Reference	Coil Structure	Size (*D_out_*_.C2_, *D_out_*_.C3_) (mm)	Freq. (MHz)	*d* (mm)	PTE %	PDL (mW), *R_load_*(Ω)
[27]	Fully Planar PSC	(50, 50)	13.56	100	77.27	-
[42]	Planar PSC	(120, 120)	50.25	100	43.62	-
[26]	Fully Planar PSC	(53, 53)	160	40	48	-
[40]	Planar PSC	(36.5, 10)	13.56	10	83.5	3.9, 100
[23]	Litz Wire	(64, 22)	0.7	20	82	75, 100
This Work	Sq. Fully Planar PSC	(65.5, 20.5)	13.56	10	79.8	396, 100
This Work	Cir. Fully Planar PSC	(65, 20.3)	13.56	10	78.43	443.5, 100

**Table 6:** Human Tissue Electrical Specification (13.56 MHz).

Tissue Type	Conductivity (S·m^−1^)	Relative Permittivity
Skin	0.38	177.13
Fat	0.03	11.83
Muscle	0.62	138.44

## References

[1] Harrison R.R., Watkins P.T., Kier R.J., Lovejoy R.O., Black D.J., Solzbacher F. (2007). A low-power integrated circuit for a wireless 100-electrode neural recording system. IEEE J. Solid State Circuits.

[2] Clark G.M. (2003). Cochlear Implants: Fundamentals and Applications.

[3] Humayun M.S., Weiland J.D., Fujii G.Y., Greenberg R., Williamson R., Little J., Mech B., Cimmarusti V., Van Boemel G., Dagnelie G. (2003). Visual perception in a blind subject with a chronic microelectronic retinal prosthesis. Vis. Res..

[4] Ghovanloo M., Atluri S. (2008). An integrated full-wave CMOS rectifier with built-in back telemetry for RFID and implantable biomedical applications. IEEE Trans. Circuits Syst. I Regul. Pap..

[5] Segura-Quijano F., García-Cantón J., Sacristán J., Osés T., Baldi A. (2008). Wireless powering of single-chip systems with integrated coil and external wire-loop resonator. Appl. Phys. Lett..

[6] Cong P., Chaimanonart N., Ko W.H., Young D. (2009). A wireless and batteryless 10-bit implantable blood pressure sensing microsystem with adaptive RF powering for real-time laboratory mice monitoring. IEEE J. Solid State Circuits.

[7] Jow U.M., Ghovanloo M. (2013). Geometrical design of a scalable overlapping planar spiral coil array to generate a homogeneous magnetic field. IEEE Trans. Magn..

[8] Liou C.Y., Kuo C.J., Lee M.L., Mao S.G. Wireless charging system of mobile handset using metamaterial-based cavity resonator. Proceedings of the Microwave Symposium.

[9] Sample A.P., Meyer D.T., Smith J.R. (2011). Analysis, experimental results, and range adaptation of magnetically coupled resonators for wireless power transfer. IEEE Trans. Ind. Electron..

[10] Choi B., Nho J., Cha H., Ahn T., Choi S. (2004). Design and implementation of low-profile contactless battery charger using planar printed circuit board windings as energy transfer device. IEEE Trans. Ind. Electron..

[11] Huh J., Lee S., Lee W., Cho G., Rim C. (2011). Narrow-width inductive power transfer system for online electric vehicles. IEEE Trans. Power Electron..

[12] Wang C.S., Stielau O.H., Covic G.A. (2005). Design considerations for a contactless electric vehicle battery charger. IEEE Trans. Ind. Electron..

[13] Baker M.W., Sarpeshkar R. (2007). Feedback analysis and design of RF power links for low-power bionic systems. IEEE Trans. Biomed. Circuits Syst..

[14] Neihart N., Harrison R. (2005). Micropower circuits for bidirectional wireless telemetry in neural recording applications. IEEE Trans. Biomed. Eng..

[15] Chen H., Liu M., Jia C., Zhang C., Wang Z. Low power IC design of the wireless monitoring system of the orthopedic implants. Proceedings of the IEEE EMBS Conference.

[16] Smith S., Tang T., Terry J. (2007). Development of a miniaturised drug delivery system with wireless power transfer and communication. Inst. Eng. Technol. Nanobiotechnol..

[17] Wang G., Liu W., Sivaprakasam M., Zhou M., Weiland J.D., Humayun M.S. A dual band wireless power and data telemetry for retinal prosthesis. Proceedings of the IEEE EMBS Conference.

[18] Ghovanloo M., Najafi K. (2007). A wireless implantable multichannel microstimulating system-on-a-chip with modular architecture. IEEE Trans. Neural Syst. Rehabil. Eng..

[19] Kilinc E.G., Dehollian C., Maloberti F. Design and optimization of inductive power transmission for implantable sensor system. Proceedings of the 11th International Workshop on Symbolic and Numerical Methods, Modeling and Application to Circuit Design (SM2ACD).

[20] Duan Z., Guo Y., Kwong D. (2012). Rectangular coils optimization for wireless power transmission. Radio Sci..

[21] Jow U.M., Ghovanloo M. (2009). Modeling and optimization of printed spiral coils in air, saline, and muscle tissue environments. IEEE Trans. Biomed. Circuits Syst..

[22] Jow U.M., Ghovanloo M. (2007). Design and optimization of printed spiral coils for efficient transcutaneous inductive power transmission. IEEE Trans. Biomed. Circuits Syst..

[23] RamRakhyani A.K., Mirabbasi S., Chiao M. (2011). Design and optimization of resonance-based efficient wireless power delivery systems for biomedical implants. IEEE Trans. Biomed. Circuits Syst..

[24] Hu H., Georgakopoulos S.V. Wireless Power Transfer in Human Tissue via Conformal Strongly Coupled Magnetic Resonance. Proceedings of the IEEE Wireless Power Transfer Conference.

[25] Jonah O., Georgakopoulos S.V., Yao S., Tentzeris M.M. Conformal device for wireless powering in biomedical application. Proceedings of the 2014 IEEE Antennas and Propagation Society International Symposium (APSURSI).

[26] Hu H., Bao K., Gibson J., Georgakopoulos S.V. Printable and conformal strongly coupled magnetic resonant systems for wireless powering. Proceedings of the IEEE Wireless and Microwave Technology Conference.

[27] Jolani M., Yu Y., Chen Z. (2014). A planar magnetically coupled resonant wireless power transfer system using printed spiral coils. IEEE Lett. Antennas Wirel. Propag..

[28] Bao K., Hu H., Georgakopoulos S.V. Design considerations of conformal SCMR system. Proceedings of the IEEE Wireless Power Transfer Conference.

[29] Finkenzeller K. (2003). RFID Handbook: Fundamentals and Applications in Contactless Smart Cards and Identification.

[30] Mohan S.S., Maria D., Hershenson M., Boyd S.P., Lee T.H. (1999). Simple accurate expressions for planar spiral inductances. IEEE J. Solid State Circuits.

[31] Raju S., Wu R., Chan M., Yue C.P. (2014). Modeling of mutual coupling between planar inductors in wireless power applications. IEEE Trans. Power Electron..

[32] Gevorgian S., Berg H., Jacobsson H., Lewin T. (2003). Basic parameters of coplanar-strip waveguides on multilayer dielectric/semiconductor substrates, Part 1: High permittivity superstrates. IEEE Microw. Mag..

[33] Pieters P., Vaesen K., Brebels S., Mahmoud S.F., DeRaedt W., Beyne E., Mertens R.P. (2001). Accurate modeling of high-Q spiral inductors in thin-film multilayer technology for wireless telecommunication applications. IEEE Trans. Microw. Theory Tech..

[34] Bahl I.J., Garg R. (1977). Simple and accurate formulas for a microstrip with finite strip thickness. IEEE Proc..

[35] Ko Y.Y., Ho S.L., Fu W.N., Zhang X. (2012). A novel hybrid resonator for wireless power delivery in bio-implantable devices. IEEE Trans. Magn..

[36] Puccetti G., Stevens C.J., Reggiani U., Sandrolini L. (2015). Experimental and numerical investigation of termination impedance effects in wireless power transfer via metamaterial. Energies.

[37] Hu H., Georgakopoulos S.V. Analysis and design of conformal SCMR WPT systems with multiple resonators. Proceedings of the 2014 IEEE Antennas and Propagation Society International Symposium (APSURSI).

[38] Solymar L., Shamonina E. (2009). Waves in Metamaterials.

[39] Kiani M., Ghovanloo M. (2012). The circuit theory behind coupled-mode magnetic resonance-based wireless power transmission. IEEE Trans. Circuits Syst. I Regul. Pap..

[40] Kiani M., Jow U.M., Ghovanloo M. (2011). Design and optimization of a 3-coil inductive link for efficient wireless power transmission. IEEE Trans. Biomed. Circuits Syst..

[41] Yi Y., Buttner U., Fan Y., Foulds I.G. 3-coil resonance based wireless power transfer system for implantable electronic. Proceedings of the IEEE Wireless Power Transfer Conference.

[42] Falavarjani M.M., Shahabadi M., Mohassel J.R. (2014). Design and implementation of compact WPT system using printed spiral resonators. IET Lett. Electron..

